# Immediate Effects of Kinesiology Taping of Quadriceps on Motor Performance after Muscle Fatigued Induction

**DOI:** 10.1155/2015/410526

**Published:** 2015-07-02

**Authors:** Ick Keun Ahn, You Lim Kim, Young-Hyeon Bae, Suk Min Lee

**Affiliations:** ^1^Department of Physical Therapy, Sahmyook University, Seoul 130-742, Republic of Korea; ^2^Department of Physical and Rehabilitation Medicine, Samsung Medical Center, Seoul 138-888, Republic of Korea; ^3^Department of Physical Therapy, Angelo State University, San Angelo, TX 76904, USA

## Abstract

*Objectives*. The purpose of this cross-sectional single-blind study was to investigate the immediate effects of Kinesiology taping of quadriceps on motor performance after muscle fatigued induction. *Design*. Randomized controlled cross-sectional design. *Subjects*. Forty-five subjects participated in this study. Participants were divided into three groups: Kinesiology taping group, placebo taping group, and nontaping group. *Methods*. Subjects performed short-term exercise for muscle fatigued induction, followed by the application of each intervention. Peak torque test, one-leg single hop test, active joint position sense test, and one-leg static balance test were carried out before and after the intervention. *Results*. Peak torque and single-leg hopping distance were significantly increased when Kinesiology taping was applied (*p* < 0.05). But there were no significant effects on active joint position sense and single-leg static balance. *Conclusions*. We proved that Kinesiology taping is effective in restoring muscle power reduced after muscle fatigued induction. Therefore, we suggest that Kinesiology taping is beneficial for fatigued muscles.

## 1. Introduction

The quadriceps muscles play an important role in controlling knee motion, providing stability, and attenuating impact loading [1–4]. While quadriceps impairment is often the focus of rehabilitation and prevention interventions, few studies address how this impairment alters neuromuscular control and the knee mechanical loading environment. Examining direct effects of decreased quadriceps torque-generating capabilities on gait biomechanics and electromyography (EMG) patterns should provide the foundation for linking the knee mechanical environment with quadriceps impairment during a fundamental task [4].

Three models have been used to explore quadriceps impairment in healthy individuals: induced pain, induced effusion, and induced fatigue [4–7]. Such fatigue-related manifestations typically present as altered proprioception, delayed muscle responses, and altered biomechanics, offering the potential for injurious changes within the resultant neuromechanical profile [8–10]. Even fatigue can limit exercise performance in activities ranging from a few seconds in length to those lasting for several hours [11–15]. Several studies showed that performing short-term fatiguing exercise for various muscles reduced proprioception and force [16–19].

The Kinesiology tape was invented by Dr. Kenso Kase from Tokyo, Japan, in the 1970s. Kase et al. proposed several taping mechanisms with various intended results depending on the characteristics of the grain and elasticity of the tape applied. And the possible mechanism of increasing proprioceptive function with Kinesiology taping (KT) was cutaneous afferent stimulation through the skin [20]. The previous KT studies showed controversial responses in clinical trial. Some studies of advantageous effects of KT were reported, such as enhancement of muscle function, improvement of joint alignment and neuromuscular performance, and reduction of lymphoedema [20–23]. However, other studies found no effects of KT on performance of various muscles in healthy subjects [24, 25]. It has also been suggested that KT does not influence muscle strength [24–26]. Furthermore, KT applied to the quadriceps did not affect one-footed static balance and performance in single-hop and triple-hop tests [25]. In this regard, a recent study showed that KT increased muscle power and muscle activity of the quadriceps induced by 50 Hz vibration [27]. Overall, there is not enough evidence of the effects of KT in healthy subjects. Therefore, the purpose of this study was to investigate the immediate effects of KT of quadriceps on motor performance after muscle fatigued induction.

## 2. Methods

### 2.1. Subjects

Forty-five healthy female volunteers participated in this study. Subjects were 25 to 40 years old. They were then screened against inclusion and exclusion criteria. Inclusion criteria were as follows: (1) they reported no contraindication to participate in all tests; (2) they provided their informed written consent; and (3) they had healthy conditions. Exclusion criteria applied during screening included (1) any surgery and broken bones in the past year; (2) reported neurologic conditions; (3) pregnancy; (4) knee joint injury or laxity, knee or hip joint muscle injuries, tendinitis, and overuse; (5) patellofemoral pain syndrome; and (6) pain on knee motion [28]. The study protocol and procedures were approved by Sahmyook University's ethics committee.

### 2.2. Procedure

This study had a randomized controlled cross-sectional design and included three interventions. A randomized intervention list was created before the study began. That list determined whether a subject would be assigned to three study groups. The interventions were Kinesiology taping (KT), placebo taping (PT), and non-Kinesiology taping (NKT). Subjects were divided into three groups (KT group, PT group, and NKT group). Prior to testing, all subjects were educated about the test protocols, and their general characteristics were recorded. During the experiments, subjects were blinded to the methods, purpose, and effects of the interventions.

### 2.3. Muscle Fatigued Induction

Subjects performed fatiguing exercise to reduce quadriceps performance. In particular, the subject was asked to lie on a bed with the hip and knee joints of their dominant leg flexed to 90° and the other leg in the resting position. The angles of the hip and knee joints were measured with a digital inclinometer (Dualer IQ digital inclinometer, JTECH Medical, Salt Lake City, USA, 2006). The subject's thigh was fixed with a sling to the side bar of the bed. The peak torque was measured with a digital manual muscle tester (MMT) (Commander Power Track II, JTECH Medical, Salt Lake City, USA, 2008) attached under the moving bar of the bed. The subject pushed on the muscle tester located over the ankle joint. After the peak torque was measured, the subject continued to push on the digital MMT with peak contractures lasting for 5 s each and 2 s breaks between them until the peak torque dropped to 50% of the initial value [29].

### 2.4. Intervention

**Figure 1:**
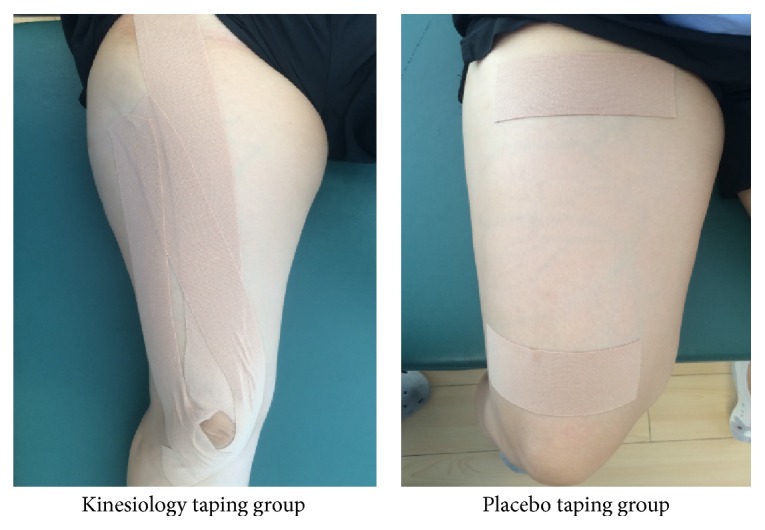
Kinesiology tape technique.

Interventions using the Kinesiology tape (BB Tape, WETAPE Inc., Seoul, Republic of Korea) were applied to the KT group and PT group by physical therapist who has KT certification with more than 10 years of experience (sport medicine). KT was applied to the quadriceps of the subjects in the KT group. The Kinesiology tape was applied with approximately 40% stretch of its maximal length on the 3 quadriceps muscles: (1) KT of rectus femoris muscle was applied from the anterior inferior iliac spine to the superior border of the patella during holding the full knee flexion; (2) KT of vastus medialis muscle was applied from the lower part of the intertrochanteric line to the medial superior aspect of the patella during holding the full knee flexion; (3) KT of vastus lateralis muscle was applied from the greater trochanter of the femur to the lateral superior aspect of the patella during holding the full knee flexion (Figure 1) [30].

In the PT group, placebo taping was applied to the muscle belly of the quadriceps without stretching from the level 5 cm below the anterior superior iliac spine (ASIS) to the level of the patella (Figure 1). Traditional Y-shape taping of the quadriceps was utilized [25, 26], and posttests were conducted immediately after the interventions in the KT and PT groups [25–27, 31, 32]. NKT group did not apply KT, and posttests were performed two minutes after the pretests because another intervention was conducted for two-minute rest. The skin over the quadriceps but not the hip and knee joints was stretched as a result of taping; this prevented quadriceps muscles stretching.

### 2.5. Measurement

#### 2.5.1. Muscle Function

Peak torque of the quadriceps was used before and after the interventions using the digital MMT for measuring muscle power. The measurement position was the same as during the muscle fatigued induction. The reliability of the quadriceps peak torque test performed with the digital MMT attached in the supine position was excellent (intraclass correlation coefficient range: 0.952–0.984) [33]. All subjects performed three isometric maximal contractions that lasted 5 s each and were separated with 30 s breaks [29]. A fatigue index was used to compare peak torque values between the groups and before and after the interventions for measuring muscle fatigue. The fatigue index was calculated according to the following formula [19]: fatigue index = (before peak torque − after peak torque)/before peak torque × 100.

#### 2.5.2. Motor Function

For single-leg hop distance measurements, the subject stood on one leg and positioned the end of the heel on a starting line on the floor. The subject then hopped forward as far as possible and landed on the same leg. Subjects were allowed to swing their arms freely and use a self-selected countermovement without stepping prior to the jump. Upon landing, subjects maintained foot contact with the floor while the investigator marked the position of the heel. This position was marked by a board marker, and the horizontal displacement in cm was measured from the heel starting line to the heel landing mark [34]. Subjects repeated the hop three times, and the mean value was used in analysis. There was a 30 s interval between the hops.

#### 2.5.3. Proprioception Function

The active joint angle reproduction (AJAR) test was used for evaluating active joint position sense (AJPS). The interrater reliability of the AJAR test is very high (ICC range: 0.91–0.99) [35, 36].

AJPS was quantified in two ways, using variable and absolute AJPS errors. Variable AJPS error is the variance of the mean constant error score and a measure of the variability of the positioning. Absolute AJPS error is the absolute value of the difference between the reproduced angle of the knee and the reference angle of the knee and is a measure of the overall accuracy of positioning [18].

The AJAR test was performed with the digital inclinometer. The subject flexed the knee to the reference angle (135°, starting angle: 90°) guided by visual feedback from a mirror. Then she reproduced the reference angle without the visual feedback three times, and the mean value was used in statistical analysis.

#### 2.5.4. Balance Function

One-footed static balance test was performed using a Wii fit board (Nintendo, Tokyo, Japan) and a static balance testing program (Balancia 2.0, Mintosys, Seocho-gu, Seoul, Republic of Korea) for measuring balance function. The subject was asked to stand on the board with the dominant leg used for support. The knee of the dominant leg was flexed to 20° as measured by the digital inclinometer. Individuals were instructed to look at a fixed point while keeping their head in the neutral position, spine erect, and upper limbs resting on the hips. The nondominant lower limb remained with the straight hip and the knee flexed to 90°. The data acquisition time was 10 seconds, and the frequency was 100 frames per second. Subjects were assessed twice, with the best result considered in analysis [25, 37].

### 2.6. Data Analysis

The statistical package SPSS 12.0 was utilized for all statistical analyses. The Kolmogorov-Smirnov (K-S) test was employed to verify the normality of data. The paired *t*-test was utilized to compare values obtained before and after the interventions in each group. After obtaining the change of difference between before and after intervention in each variable, one-way analysis of variance (ANOVA) with change of difference was performed to identify differences between the groups. The significance level was set to *p* < 0.05.

## 3. Results

The 45 subjects enrolled in this study were divided into KT, PT, and NKT groups, with 15 subjects in each group. There were no significant differences in the baseline values between the groups. The peak torque of the quadriceps was reduced after muscle fatigued induction in all groups (Table 1).

The interventions significantly changed the peak torque of the fatigued quadriceps and fatigue index in the KT and PT groups (*p* < 0.05). In contrast, there were no significant changes in the NKT group. The peak torque of the fatigued quadriceps and the fatigue index in the KT group were significantly higher than in the PT and NKT groups (*p* < 0.05) (Table 2). 

The single-leg hop distance after muscle fatigued induction was significantly altered by the interventions in the KT and PT groups (*p* < 0.05). No significant differences were observed in the NKT group. The single-leg hop distance values after muscle fatigued induction were significantly greater in the KT group than in the PT and NKT groups (*p* < 0.05) (Table 3).

There were no significant differences in the relative and absolute position sense error of the knee joint. Furthermore, no differences in the relative and absolute position sense errors of the knee joint were detected between the groups (Tables 4 [Table tab5]–6). The performance in the one-footed static balance test was not affected by muscle fatigued induction. Moreover, there were no significant differences between the groups in the results of this test (Table 7).

## 4. Discussion

The results of this study suggest that KT of quadriceps after muscle fatigued induction increases the peak torque and one-leg hop distance but does not alter the position sense and one-footed static balance.

The preceding studies showed that KT does not affect the performance of various muscles in healthy subjects [24–26, 31, 32]. In contrast, according to another report, KT significantly improved the peak torque and active EMG of the quadriceps reduced by tactile vibration stimulation [27]. Muscle fatigued induction can reduce the muscle performance in a manner similar to tactile vibration stimulation. Thus, the size of the H-reflex decreased during contracture of muscle at both maximal and submaximal target forces [38–41]. This decrease was not due to a reduction in motor neuron excitability but rather represented modulation of the afferent feedback to the motor neuron pool [41, 42]. In addition to a decrease in Ia-afferent input, the evoked responses also indicated that there is a gradual depression of spinal excitability during muscle fatigued induction. The depression of spinal cord excitability, however, appears to be paradoxically accompanied by an increase in cortical excitability [42]. These findings indicate that the voluntary activation of muscle can be impaired during muscle fatigued induction and that the depression of activity in the spinal cord is a major contributor to the deficit [41].

In this study, the muscle fatigued induction was performed until the peak torque of the quadriceps decreased to <50% of the normal value. The values of the peak torque were then compared after applying 40% stretched Kinesiology tape, placebo tape, or no tape to the quadriceps. As a result, the values in the KT group were significantly increased compared with the other two groups (*p* < 0.05). Therefore, KT effectively restored the muscle force reduced by muscle fatigued induction.

Single-leg hop distances were also compared after applying 40% stretched Kinesiology tape, placebo tape, or no tape to the quadriceps. The subjects in the KT group performed significantly better in this test than the individuals in the other two groups (*p* < 0.05). This confirms the efficiency of KT in restoring the muscle force reduced by muscle fatigued induction.

In this study, 40% stretched Kinesiology tape was applied to the stretched skin of the quadriceps using stretching motions. According to a previous report, the application of tape over the stretched skin facilitated motor function through cutaneous afferent stimulation [43]. In particular, KT applied over stretched skin stimulates the cutaneous afferent nerve, engaging *α*-motor neurons. This improves the performance of the quadriceps after muscle fatigued induction [43].

The post hoc analysis revealed that the values of peak torque and single-hop distance in the KT group were significantly greater than in the other two groups. Furthermore, there were no differences between the PT and NKT groups. In contrast, the PT group performed better than the NKT group when the average values of peak torque and single-leg hop distance were compared. Although the values in the PT group were significantly increased after the intervention, a placebo effect is unlikely. These results agree with the data of Fratocchi et al. [31].

The muscle spindle plays an important role in active joint position sense, whereas muscle receptors are important for static position balance. In this study, wrapping the quadriceps skin with Kinesiology tape targeted the muscle receptors. However, KT had no effect on the active position sense and one-footed static balance in the subjects with the quadriceps fatigued by muscle fatigued induction. This agrees with the results of Akbaş et al. and Lins et al. In particular, Akbaş et al. tested the effect of Kinesiology taping of the quadriceps on active joint position sense of the knee joint in patients with patellofemoral pain syndrome [44], whereas Lins et al. tested one-footed static balance in healthy subjects with Kinesiology tape applied to the quadriceps [25]. Another study showed that KT of the forearm affected the force sense in healthy athletic individuals [32]. Force sense is a proprioceptive sense that relies on the muscle spindle. Therefore, the study shows that KT affects muscle receptors [32].

In this study, PT did not influence proprioception, which depends on muscle receptors. This confirms that proprioceptive sense was not affected by placebo effects.

It is not clear at present whether KT affects muscle receptors. The results of this study suggest two possible explanations as to why KT failed to stimulate these receptors. First, the material of the Kinesiology tape may have been inadequate for the task. Second, the KT method commonly used in the clinics may be inappropriate to stimulate the muscle receptors. More suitable materials or taping methods must be sought in future studies.

The first limitation of this study is that long-term effects of KT were not investigated, as the tests were conducted immediately after applying the tape. Second, the relatively low number of subjects makes it difficult to generalize the results. Finally, the material of the placebo tape was different from the material of Kinesiology tape, which may have interfered with the single-blinded design of the study.

Therefore, the mechanism underlying the effects of KT on fatigue muscle is not enough. Further studies are required to resolve the limitations of the current study and should consider various methods of Kinesiology tape application, include more subjects, and address the difference in the materials of Kinesiology tape and placebo tape.

## 5. Conclusion

In this study, peak torque, single-leg hop distance, active position sense, and one-footed static balance tests were used to assess the effects of KT in 45 healthy subjects whose quadriceps were fatigued by muscle fatigued induction. The KT group performed significantly better than the PT and NKT groups in terms of peak torque and single-leg hop distance (*p* < 0.000). This suggests that KT is an efficient method for reducing muscle fatigue.

## Figures and Tables

**Table 1:** General characteristics of the subjects.

	KT group	PT group	NKT group
Gender (male/female)	15 (0/15)	15 (0/15)	15 (0/15)
Age (years)	29.06 ± 2.84	31.33 ± 4.11	30.26 ± 3.41
Height (cm)	161.33 ± 4.30	161.26 ± 3.17	161.53 ± 5.70
Weight (kg)	54.73 ± 5.61	54.26 ± 6.43	52.33 ± 3.30
Dominant leg (right/left)	15/0	15/0	15/0

Note: values are expressed as means ± standard deviation (SD). No significant difference.

**Table 2:** Quadriceps peak torque before and after the interventions.

		KT group	PT group	NKT group
Peak torque (N·m)	Before fatiguing exercise	144.48 ± 36.41	138.28 ± 33.11	136.77 ± 30.59
	Before intervention	71.56 ± 18.52	66.24 ± 16.04	68.33 ± 16.99
	After intervention	111.35 ± 31.58	72.64 ± 16.18	70.00 ± 16.64
	Difference	39.79 ± 18.27^*∗*#^	6.76 ± 1.83^*∗*^	4.09 ± 9.57

Fatigue index (score)	Before intervention Before fatiguing exercise	50.53 ± 1.29	51.80 ± 1.04	52.02 ± 1.34
	After intervention Before intervention	27.91 ± 11.25^*∗*#^	3.80 ± 2.32^*∗*^	2.30 ± 3.52

Note: values are expressed as means ± standard deviation (SD). ^*∗*^Significant difference between the values before and after in the each group. ^#^Significant difference between the KT group and the other groups (*p* < 0.05).

**Table 3:** Single-leg hop distance before and after the interventions.

		KT group	PT group	NKT group
Single-leg hop distance (cm)	Before intervention	76.86 ± 18.22	64.94 ± 13.55	79.80 ± 19.40
	After intervention	85.33 ± 18.46	67.06 ± 14.05	78.66 ± 19.24
	Difference	8.46 ± 6.19^*∗*#^	2.13 ± 1.59^*∗*^	1.13 ± 3.09

Note: values are expressed as means ± standard deviation (SD). ^*∗*^Significant difference between the values before and after in the each group. ^#^Significant difference between the KT group and the other groups (*p* < 0.05).

**Table 4:** Active joint position sense before and after the interventions (reproduced angle).

		KT group	PT group	NKT group
Reproduced angle (°)	Before intervention	76.86 ± 18.22	64.94 ± 13.55	79.80 ± 19.40
	After intervention	85.33 ± 18.46	67.06 ± 14.05	78.66 ± 19.24
	Difference	8.46 ± 6.19^*∗*#^	2.13 ± 1.59^*∗*^	1.13 ± 3.09^*∗*^

Note: values are expressed as means ± standard deviation (SD). ^*∗*^No significant difference between the values before and after the interventions. ^#^No significant difference between the KT group and the other groups.

**Table 5 tab5:** Relative active joint position sense error before and after the interventions.

		KT group	PT group	NKT group
Relative AJPS error (°)	Before intervention	2.80 ± 4.53	2.06 ± 5.25	2.77 ± 4.47
	After intervention	3.00 ± 4.33	2.40 ± 5.11	2.88 ± 4.27
	Difference	0.20 ± 4.25^*∗*#^	0.33 ± 2.16^*∗*^	0.11 ± 0.69^*∗*^

Note: values are expressed as means ± standard deviation (SD). ^*∗*^No significant difference between the values before and after the intervention. ^#^No significant difference between the KT group and the other groups.

**Table 6:** Absolute active joint position sense error before and after the interventions.

		KT group	PT group	NKT group
Absolute AJPS error (°)	Before intervention	4.93 ± 1.69	5.35 ± 1.21	5.08 ± 0.76
	After intervention	4.82 ± 1.86	5.20 ± 1.82	4.97 ± 0.80
	Difference	0.11 ± 2.24^*∗*#^	0.15 ± 1.99^*∗*^	0.11 ± 0.69^*∗*^

Note: values are expressed as means ± standard deviation (SD). ^*∗*^No significant difference between the values before and after the interventions. ^#^No significant difference between the KT group and the other groups.

**Table 7:** One-footed static balance before and after the interventions.

One-footed static balance		KT group	PT group	NKT group
Velocity of the center of gravity (mm/sec)	Before intervention	4.80 ± 0.40	4.89 ± 0.45	4.82 ± 0.42
	After intervention	4.78 ± 0.37	4.87 ± 0.45	4.83 ± 0.41
	Difference	0.02 ± 0.03^*∗*#^	0.02 ± 0.03^*∗*^	0.01 ± 0.04^*∗*^

Distance from the center of gravity (mm)	Before intervention	48.07 ± 4.08	49.01 ± 4.50	48.26 ± 4.30
	After intervention	47.83 ± 3.72	48.80 ± 4.51	48.42 ± 4.18
	Difference	0.24 ± 0.32^*∗*#^	0.21 ± 0.37^*∗*^	0.16 ± 0.42^*∗*^

Note: values are expressed as means ± standard deviation (SD). ^*∗*^No significant difference between the values before and after the intervention. ^#^No significant difference between the KT group and the other groups.
